# Comparative transcriptome and miRNA analysis of skin pigmentation during embryonic development of Chinese soft-shelled turtle (*Pelodiscus sinensis*)

**DOI:** 10.1186/s12864-022-09029-y

**Published:** 2022-12-05

**Authors:** Pei Wang, Gang Xiong, Dan Zeng, Jianguo Zhang, Lingrui Ge, Li Liu, Xiaoqing Wang, Yazhou Hu

**Affiliations:** 1 College of Animal Science and Technology, Hunan Agricultural University, Changsha, 410128 China; 2 Hunan Biological and Electromechanical Polytechnic, Changsha, 410127 Hunan China; 3 College of Life and Environmental Science, Hunan University of Arts and Science, Changde, 415000 Hunan China; 4 School of Medical Technology, Shaoyang University, Shaoyang, 422000 Hunan China

**Keywords:** Pelodiscus sinensis, Transcriptome, microRNA, Embryonic development, Melanogenesis

## Abstract

**Background:**

Aquatic animals show diverse body coloration, and the formation of animal body colour is a complicated process. Increasing evidence has shown that microRNAs (miRNAs) play important regulatory roles in many life processes. The role of miRNAs in pigmentation has been investigated in some species. However, the regulatory patterns of miRNAs in reptile pigmentation remain to be elucidated. In this study, we performed an integrated analysis of miRNA and mRNA expression profiles to explore corresponding regulatory patterns in embryonic body colour formation in the soft-shelled turtle *Pelodiscus sinensis*.

**Results:**

We identified 8 866 novel genes and 9 061 mature miRNAs in the skin of Chinese soft-shelled turtles in three embryonic stages (initial period: IP, middle period: MP, final period: FP). A total of 16 563 target genes of the miRNAs were identified. Furthermore, we identified 2 867, 1 840 and 4 290 different expression genes (DEGs) and 227, 158 and 678 different expression miRNAs (DEMs) in IP *vs.* MP, MP *vs.* FP, and IP *vs.* FP, respectively. Among which 72 genes and 25 miRNAs may be related to turtle pigmentation in embryonic development. Further analysis of the novel miRNA families revealed that some novel miRNAs related to pigmentation belong to the miR-7386, miR-138, miR-19 and miR-129 families. Novel_miR_2622 and novel_miR_2173 belong to the miR-19 family and target *Kit* and *Gpnmb*, respectively. The quantification of novel_miR_2622 and *Kit* revealed negative regulation, indicating that novel_miR_2622 may participate in embryonic pigmentation in *P. sinensis* by negatively regulating the expression of *Kit*.

**Conclusions:**

miRNA act as master regulators of biological processes by controlling the expression of mRNAs. Considering their importance, the identified miRNAs and their target genes in Chinese soft-shelled turtle might be useful for investigating the molecular processes involved in pigmentation. All the results of this study may aid in the improvement of *P. sinensis* breeding traits for aquaculture.

**Supplementary Information:**

The online version contains supplementary material available at 10.1186/s12864-022-09029-y.

## Background

Skin colouration is an important phenotypic trait with exerts multiple adaptive functions and functions in biological processes, including roles in species identification, thermoregulation, camouflage, warning or threatening predators, social communication, and selective mating [1–3]. Both external factors (environment, nutritional) and internal factors (genetic, cellular, nervous, hormonal) [4] can affect skin pigmentation in animals. To date, melanogenesis is the most thoroughly elucidated signalling pathway in pigmentation research. The complex process of melanogenesis involves many genes, such as those encoding tyrosinases (*Tyr*), tyrosinase-related protein 1 (*Tyrp1*), dopachrome tautomerase (*Dct*), solute carrier family 45 member 2 (*Slc45a2*), and G-protein coupled receptor 143 (*Gpr143*), which are the major regulators [5]. Moreover, SRY-box transcription factor 10 (*Sox10*) and KIT proto-oncogene receptor tyrosine kinase (*Kit*), and the most critical gene that affects the production of *Tyr*, *Tyrp1* and *Dct* is microphthalmia-associated transcription factor (*Mitf*) [6–9]. To elucidate the molecular mechanisms of fish skin variation and the genetic basis of pigmentation, extensive studies have been conducted on model fishes, such as zebrafish (*Barchydanio rerio* var) [10, 11] and medaka (*Oryzias latipes*) [12, 13]. More recently, body colour-related genes have been studied in an increasing number of nonmode aquatic animals. *Spr* was reported to be associated with the differentiation and formation of xanthophores/erythrophores in Japanese ornamental carp (*Cyprinus carpio* var. *koi*) [14]. Ahi Ehsan Pashay et al. revealed *Bco2* as a candidate carotenoid colour gene via comparative transcriptomics [15]. Lu et al. established *csf1ra* mutant lines with two different targets by CRISPR/Cas9 in Nile tilapia (*Oreochromis niloticus*), and they found that *csf1ra* activity was essential for the development of erythrophores, late-developing xanthophores and late metamorphic melanophores [16].

MicroRNAs (miRNAs) are a type of single-stranded, noncoding RNA molecules with an average size of approximately 22 nucleotides. Generally, miRNAs bind to 3’ or 5’—untranslated regions (UTRs) of mRNAs and block the translation of genes or the induce cleavage of mRNAs, which is consistent with their effect on the posttranscriptional regulation of protein expression [17, 18]. As an important posttranscriptional regulator, miRNAs also play pivotal roles in animal skin colouration by regulating pigmentation gene expression. For example, in humans, miRNA-203 regulates melanosome transport and tyrosinase expression in melanoma cells by targeting kinesin superfamily protein 5b (*Kif5b*) [19]. MiRNA-218 can inhibit TYR activity and the trigger decolorization of murine immortalized melanocytes by directly targeting *Mitf* and inducing melanogenesis disorders [20]. The expression level of miR-137 can affect the body colour pattern in mice [21]. MiRNA-429 has been reported to be related to common carp (*Cyprinus carpio* var. *color*) skin colour, and its silencing can increase *Foxd3* expression, which inhibits *Mitf* transcription [22]. In *Botia superciliaris*, the overexpression of miR-217-5p can inhibit pheomelanin formation by targeting *Gsta2* (*Zgc*) [23]. Although transcriptomic investigations of variations in skin colour have been conducted in common carp [24], chicken (*Gallus gallus*) [25], red tilapia (*Oreochromis mossambicus*) [26], red crucian carp (*Carassius auratus*, red var.) [27], and Japanese flounder (*Paralichthys olivaceus*) [28], few studies have checked into this topic in reptiles to date.

Chinese soft-shelled turtle (*Pelodicus sinensis*), is an important commercial freshwater reptile, that is cultured in many Asian countries, such as China, Korea, Japan, and Vietnam [29, 30]. In China, it is considered not only to be a rich source of flavourful protein, but also to have medicinal value. In recent years, the production of *P. sinensis* has increased rapidly, and it has become one of the largest-scale reptile industries in China, with a total culture yield of 332 616 tons in 2020 [31]. Similar to other aquatic animals, the body colour of Chinese soft-shelled turtle is one of the traits worth paying attention to. For example, Qingxi black turtle and Yongzhang golden turtle are two new varieties of Chinese soft-shelled turtle bred with body color as the breeding goal, and they are very popular among consumers. Although the body colour of Chinese soft-shelled turtle is affected by environmental [32] and nutritional factors [33], the genetic factors are still the most important [34]. To date, more than 38 000 miRNAs found in animals and plants have been identified and deposited in the miRNA registry database (miRBase Release 22.1, October 2018) (http://www.mirbase.org/), but there are no miRNAs of *P. sinensis*. Research on turtle miRNAs has focused on sex regulation [35, 36], evolution [37, 38], lipid metabolism [39, 40], and miRNA identification [41]. To better understand the mechanisms underlying skin colour formation in *P. sinensis*, we used high-throughput sequencing technologies to screen differentially expressed mRNAs and miRNAs in three embryonic development stages of Chinese soft-shelled turtle. Several mRNAs and miRNAs were identified as candidates that may regulate body colour formation in *P. sinensis* skin. This study reveals the mechanism of skin colour formation in *P. sinensis* at the mRNA and miRNA transcriptome levels, and provides basic information for further elucidating the genetic mechanisms of melanogenesis and reproduction in aquatic animals.

## Results

### Overview of RNA-seq data

**Fig. 1:**
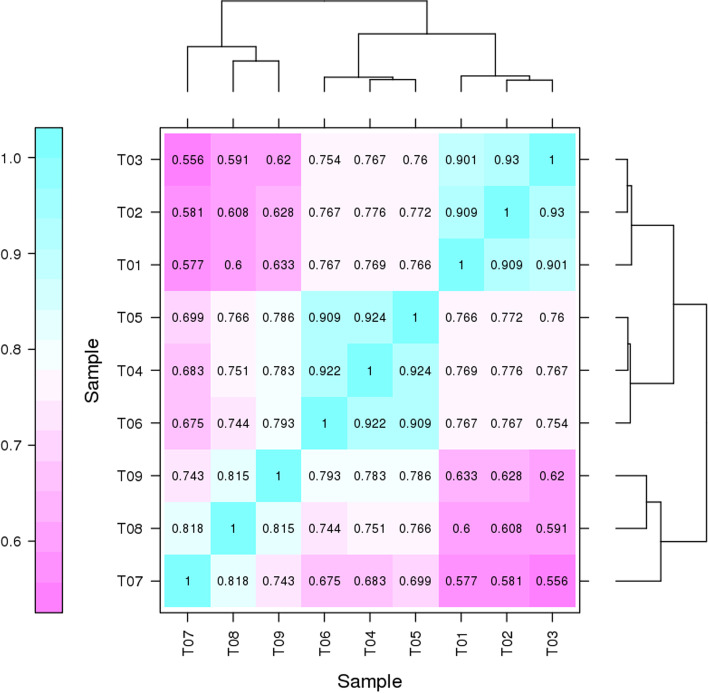
Pearson correlation coefficient and hierarchical clustering analysis based on FPKM values of skirt tissue transcripts of Chinese soft-shelled turtle in three incubation periods. The numbers represent Pearson correlation coefficients. Purple indicates a low correlation coefficient, and blue indicates a high correlation coefficient

In this study, 9 cDNA libraries from hatching turtle embryo were established and sequenced. After filtering, the total number of clean reads per library were ranged from 40.94 to 53.85 million. The quality assessment of the sequencing data showed that more than 93.62% of the reads from each sample had a quality score of Q30. The GC content and AT content were almost equal in each sequencing cycle. Subsequently, 79.46–82.90% of the clean reads were successfully mapped to the *Pelodiscus sinensis* genome, and 75.54–79.83% of the reads were uniquely mapped to the genome in the three hatching stages (Table 1). A total of 8 866 novel genes were discovered, and 5 019 genes were annotated via BLAST according to COG, GO, KEGG, KOG, Pfam, Swiss-Prot, EggNOG, and NR database (Table 2). These results demonstrated that the sequencing quality was quite good, and that the subsequent transcriptome analysis results were therefore reliable. Based on the expression level of each gene in the samples, the correlation coefficients were calculated to evaluate repeatability. The results showed that the Pearson correlation coefficient among the individuals from IP and MP was larger than 0.9, indicating that biological replicates were highly correlated (Fig. 1). In the hierarchical clustering analysis, the IP and MP samples were first clustered together and then clustered together with the FP samples. The results showed that the expression patterns of skirt transcripts were similar in the initial and middle periods, but different from those in the final period.

**Table 1:** Statistical analysis of transcriptome sequencing data

Group	Sample	Clean reads	GC content (%)	% ≥ Q30(%)	Total reads	Mapped reads (%)	Unique mapped reads (%)	Multiple mapped reads (%)
Initial Period	T01	20 848 242	50.85	93.77	41 696 484	34 056 360 (81.68%)	32 722 981 (78.48%)	1 333 389 (3.20%)
	T02	20 474 011	50.54	93.75	40 948 022	33 677 763 (82.25%)	32 395 320 (79.11%)	1 282 442 (3.13%)
	T03	23 332 118	50.10	94.35	46 664 236	38 683 111 (82.90%)	37 251 411 (79.83%)	1 431 700 (3.07%)
Middle Period	T04	26 151 645	51.21	93.66	53 303 290	42 550 588 (81.35%)	40 728 455 (77.87%)	1 822 133 (3.48%)
	T05	26 927 213	51.19	93.91	53 854 426	43 130 689 (80.09%)	41 314 852 (76.72%)	1 815 837 (3.37%)
	T06	21 680 460	50.99	93.62	43 360 920	34 770 201 (80.19%)	33 325 548 (76.86%)	1 444 653 (3.33%)
Final Period	T07	20 733 788	51.97	94.03	41 467 576	33 140 221 (79.19%)	31 655 038 (76.34%)	1 485 183 (3.58%)
	T08	21 866 869	51.61	93.71	43 733 738	35 213 233 (80.52%)	33 612 941 (76.86%)	1 600 292 (3.66%)
	T09	21 670 052	51.81	93.97	43 340 104	34 440 131 (79.46%)	32 740 685 (75.54%)	1 699 446 (3.92%)

**Table 2:** Statistical annotation of new genes

Annotated Databases	COG	GO	KEGG	KOG	Pfam	Swiss-Prot	EggNOG	Nr	All
New Gene Number	387	2 504	1 820	2 026	1 937	1 431	4 010	4 896	5 019

### Differentially expressed gene identification and functional enrichment analyses

**Fig. 2:**
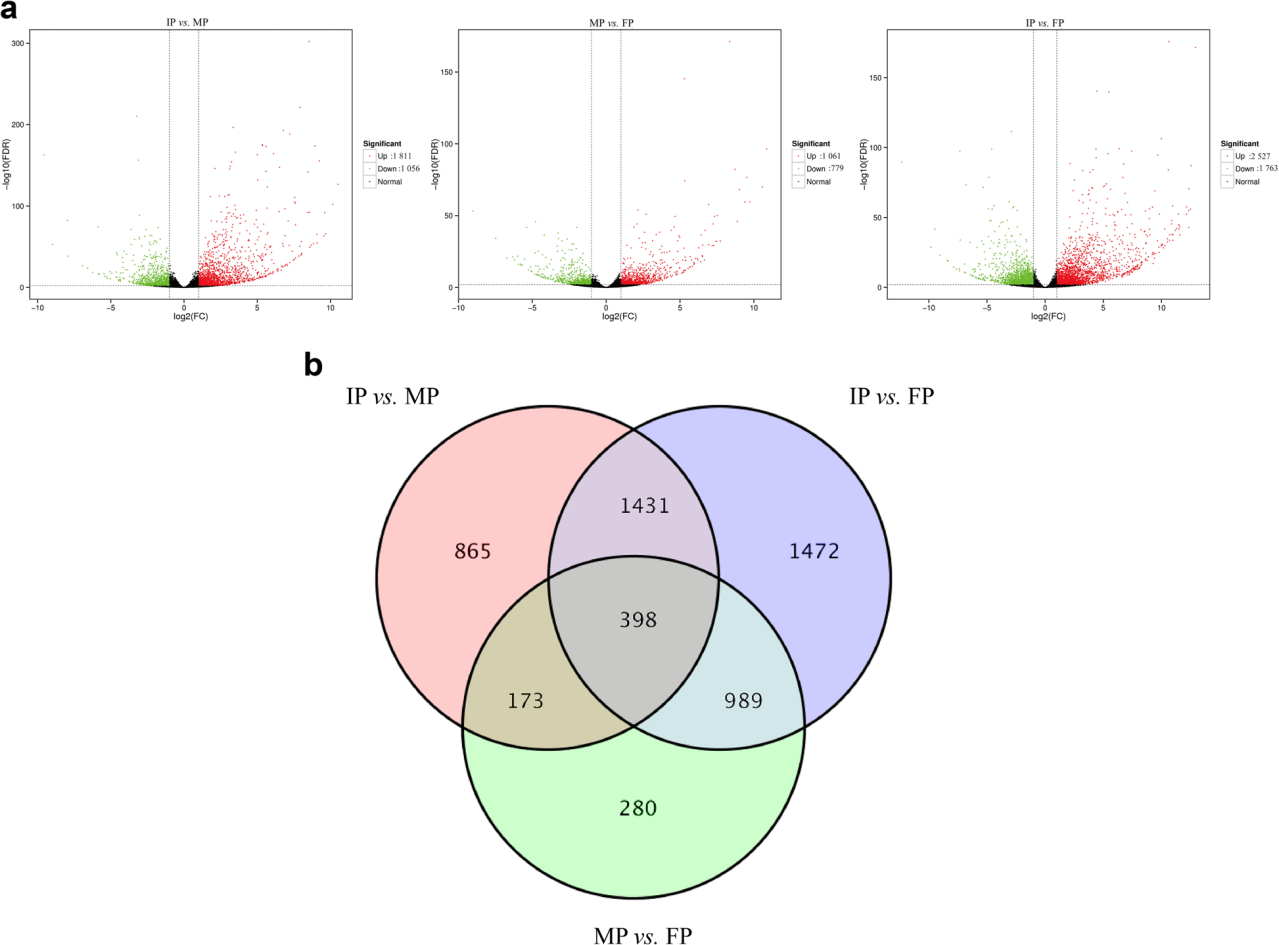
Analysis of differentially expressed genes in different incubation periods. **a**. Volcano plot of differentially expressed genes in the three periods; **b**. Venn map of pairwise comparison in three periods

**Fig. 3:**
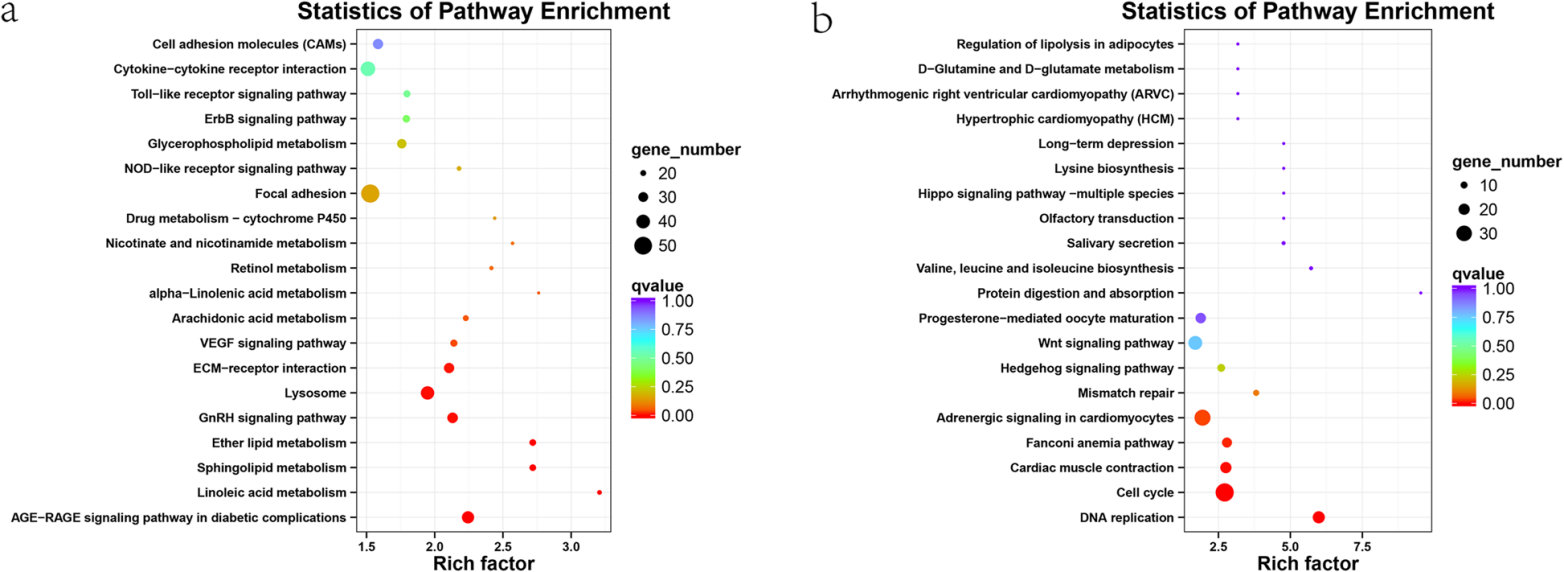
KEGG enrichment of Veen genes. **a**. Upregulated genes, **b**. downregulated genes. The X-axis shows the enrichment score. The larger the number of different genes is, the larger the bubble. Bubble colour ranges from red to blue, and the enrichment q-value is large

To compare differential expression patterns among IP, MP, and FP, DEGs were identified using DEseq2 software with a corrected *q-value* < 0.01 and a fold change (FC) ≥ 2. The subsequent differential expression analysis showed that 2 867 DEGs were detected in IP *vs.* MP, among which 1 811 DEGs were upregulated and 1 056 were downregulated in IP. In MP *vs.* FP, a total of 1 840 genes were differentially expressed, among which 1 061 were upregulated in MP, and 779 were downregulated compared with their levels in the final period. A total of 4 290 transcripts were identified as DEGs in IP *vs.* FP, which 2 527 and 1 763 DEGs were significantly upregulated and downregulated, respectively (Fig. 2a, Tab. S1). Further analysis indicated that a total of 398 genes showed significantly different expression levels in the three comparison groups (Fig. 2b, Tab. S2). Among which 258 genes were only upregulated and 96 genes were only downregulated. These up/downregulated differential genes were significantly enriched in linoleic acid metabolism, adrenergic signalling in cardiomyocytes, apoptosis, and the gap junction pathway (*p-value* < 0.001) (Fig. 3).

**Fig. 4:**
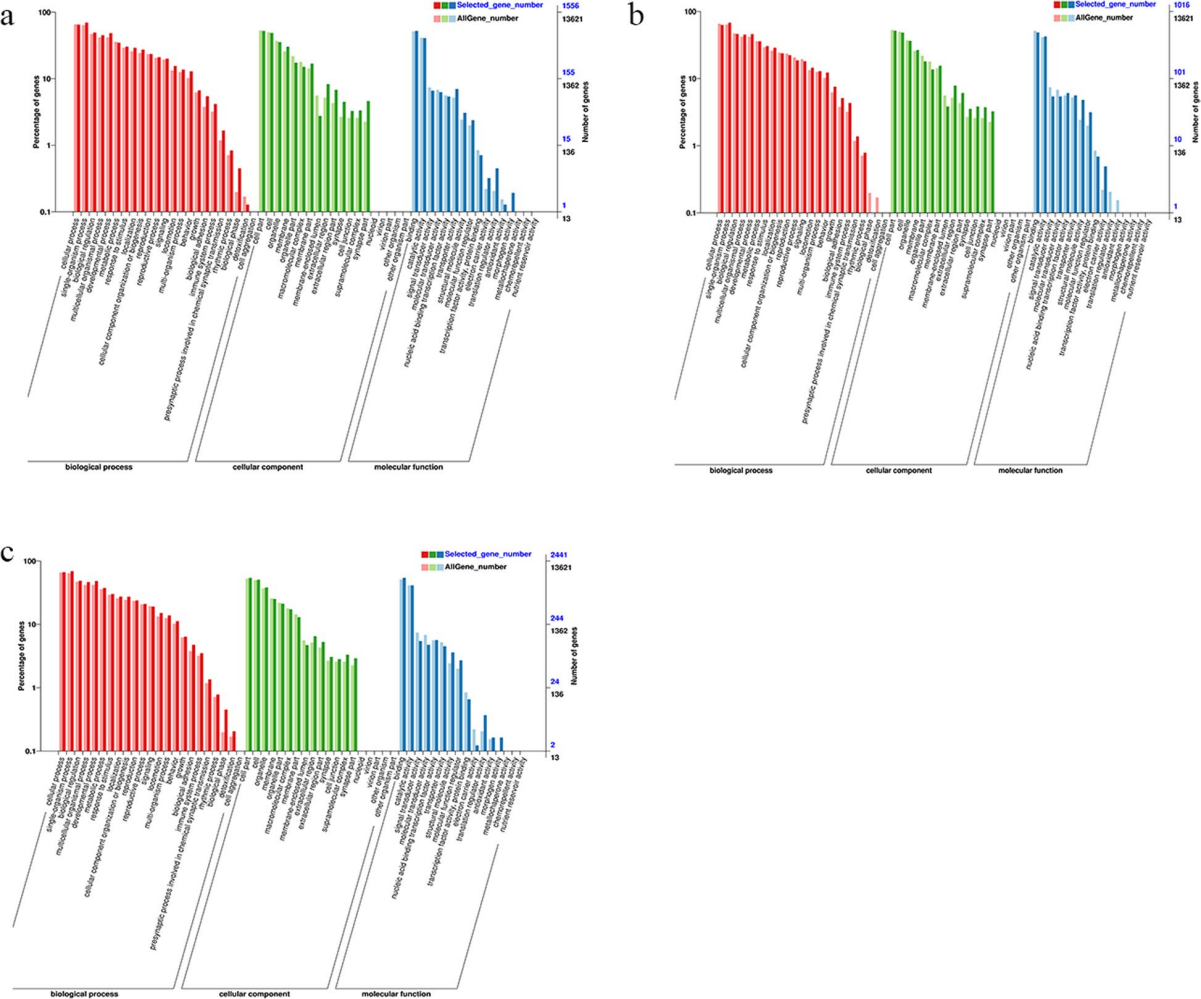
Gene Ontology (GO) functional classification of differentially expressed genes (DEGs) in P. sinensis. a: IP vs. MP; b: MP vs. FP; c: IP vs. FP. The x-axis shows three terms, and the y-axis shows the proportions of DEGs and total genes corresponding to each subcategory. The red column represents the annotation of all genes, while the blue column represents the annotation of DEGs

The DEGs were assigned to various GO terms to determine their functional classifications. In the biological process category, single-organism process was the most abundant GO term, while in the cellular component and molecular function categories, cell and bind were the most enriched terms, respectively (Fig. 4). The GO term classifications were similar among the three groups, with little variation in molecular function. Within the biological process category, single-organism process, cellular process, and biological regulation were the dominant annotated DEGs. In the cellular component category, many DEGs were enriched in the cell part and cell. The majority of the DEGs in the molecular function category were involved in binding. GO term enrichment analysis revealed that 551, 553 and 564 GO terms with *p-values* < 0.05 were enriched in IP *vs.* MP, MP *vs.* FP and IP *vs.* FP, respectively (Tab. S3). Among these terms, the G-protein coupled receptor signalling pathway, multicellular organism development and nematode larval development were significantly enriched in the three incubation stages.

**Fig. 5:**
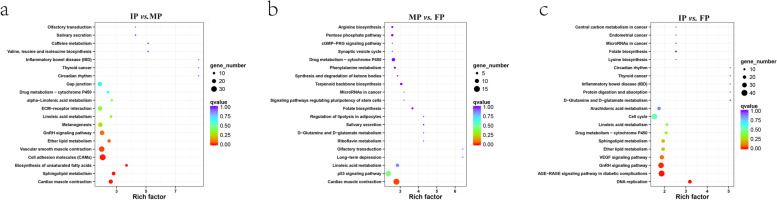
KEGG enrichment top 20 bubble chart. The X-axis shows the enrichment score. The larger the number of different genes is, the larger the bubble. Bubble colour ranges from red to blue, and the enrichment q-value is large

Annotated pathways of DEGs were analysed using the KEGG database to identify the functions of all DEGs. There were 168, 157 and 183 pathways found in IP *vs.* MP, MP *vs.* FP and IP *vs.* FP, respectively. The KEGG pathway enrichment analysis showed that 5, 1, and 3 pathways with *Q*-values < 0.05 were significantly enriched in IP *vs.* MP, MP *vs.* FP and IP *vs.* FP, respectively. The top 20 pathways with the most significant enrichment are shown in Fig. 5. KEGG analysis indicated that linoleic acid metabolism, ECM-receptor interaction, and drug metabolism—cytochrome P450 were commonly enriched signalling pathways in the three periods. Additionally, melanogenesis, cell adhesion molecules (CAMs) and the ErbB signalling pathway were also included in the top 20 pathways in IP *vs.* MP and IP *vs.* FP, respectively. The results suggested that these pathways may be involved in embryonic skin pigmentation of turtles.

### Critical DEGs involved in pigmentation in embryo of P. sinensis

**Fig. 6:**
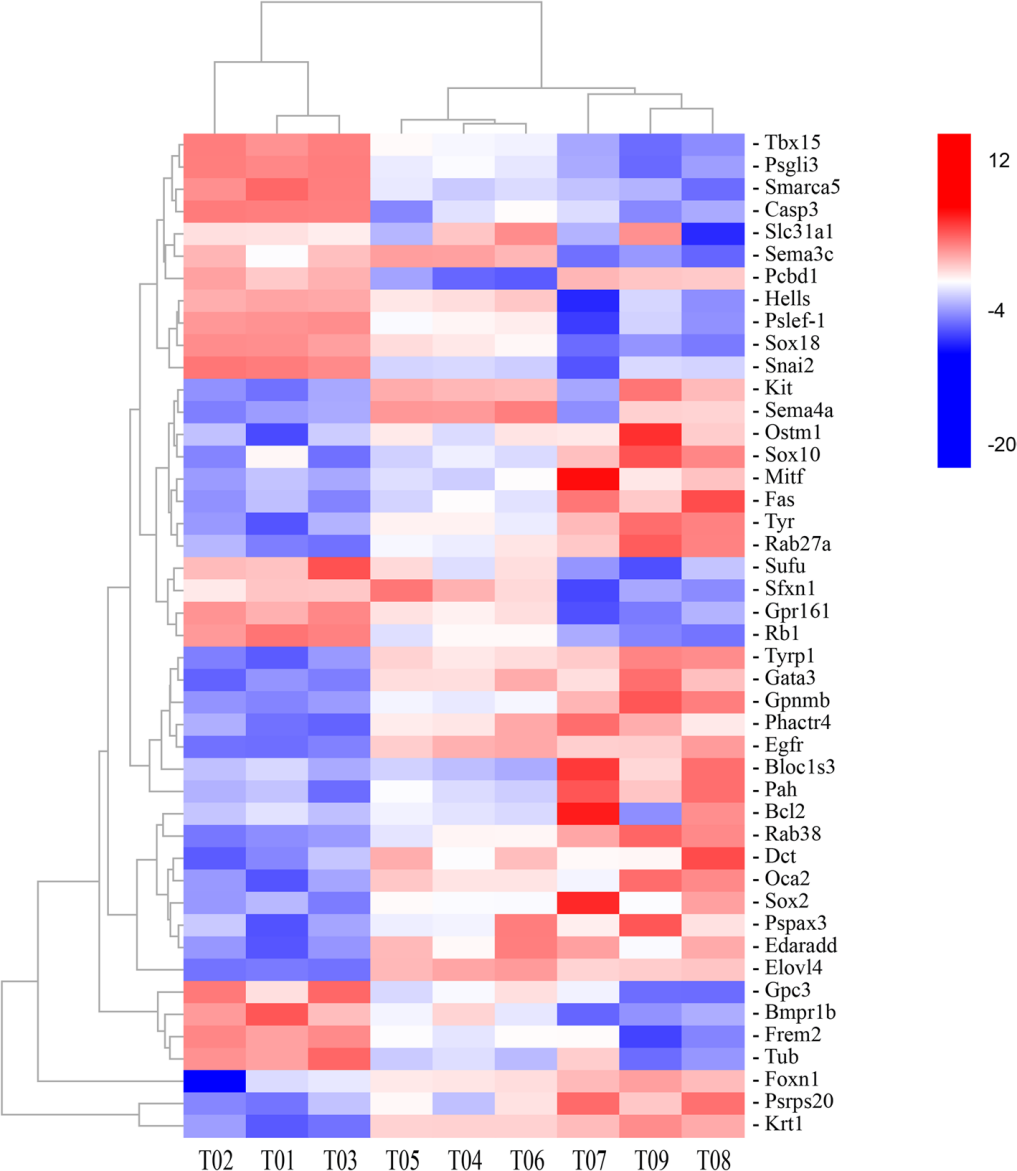
Expression patterns of differentially expressed genes related to pigmentation. Blue to red colours indicate expression values ranging from low to high

To identify the DEGs involved in pigment formation in embryos of *P. sinensis*, the KEGG results related to melanogenesis was studied in detail. In IP *vs.* MP, seven DEGs involved in the melanogenesis pathway were downregulated, and sixteen DEGs were upregulated. In MP *vs.* FP, four of the upregulated DEGs involved in melanogenesis pathways were *Mitf*, segment polarity protein dishevelled homo log DVL-1 (*Dvl1*), *Tyr*, and calmodulin like 3 (*Calml3*). While wnt family member 11 (*Wnt11*), wnt family member 5B (*Wnt5b*), and cAMP-responsive element binding protein 3-like 1 (*Creb3l1*) were downregulated. In IP *vs.* FP, there were 26 DEGs including sixteen upregulated genes and ten downregulated genes (Tab. S4). To further confirm the fidelity of the differentially expressed genes presented in this study, we compared the overlapping pigmentation related genes between previous publications (http://www.espcr.org/micemut/#cloned) and the present study. The comparison revealed 45 genes, including *Mitf*, *Sox10*, *Tyr*, *Tyrp1*, *Kit*, *Dct*, RAB27a, member RAS oncogene family (*Rab27a*), etc. Among these genes, Osteoclastogenesis associated transmembrane protein 1 (*Ostm1*), *Sox10*, *Mitf*, Fas cell surface death receptor (*Fas*), *Tyr*, *Rab27a*, *Tyrp1*, *Gpnmb*, biogenesis of lysosomal organelles complex 1 subunit 3 (*Bloc1s3*), phenylalanine hydroxylase (*Pah*), BCL2 apoptosis regulator (*Bcl2*), RAB38, member RAS oncogene family (*Rab38*), *Dct* and OCA2 melanosomal transmembrane protein (*Oca2*) were showed upregulated expression and increasing trend patterns in the three incubation stages (Fig. 6). The Combination of the DEGs of the melanogenesis pathways and the reported body colour genes resulted in a group of 72 candidate genes for embryonic pigmentation in Chinese soft-shelled turtle were obtained (Tab. S5).

### MiRNA sequencing analysis of P. sinensis skin tissue

**Fig. 7:**
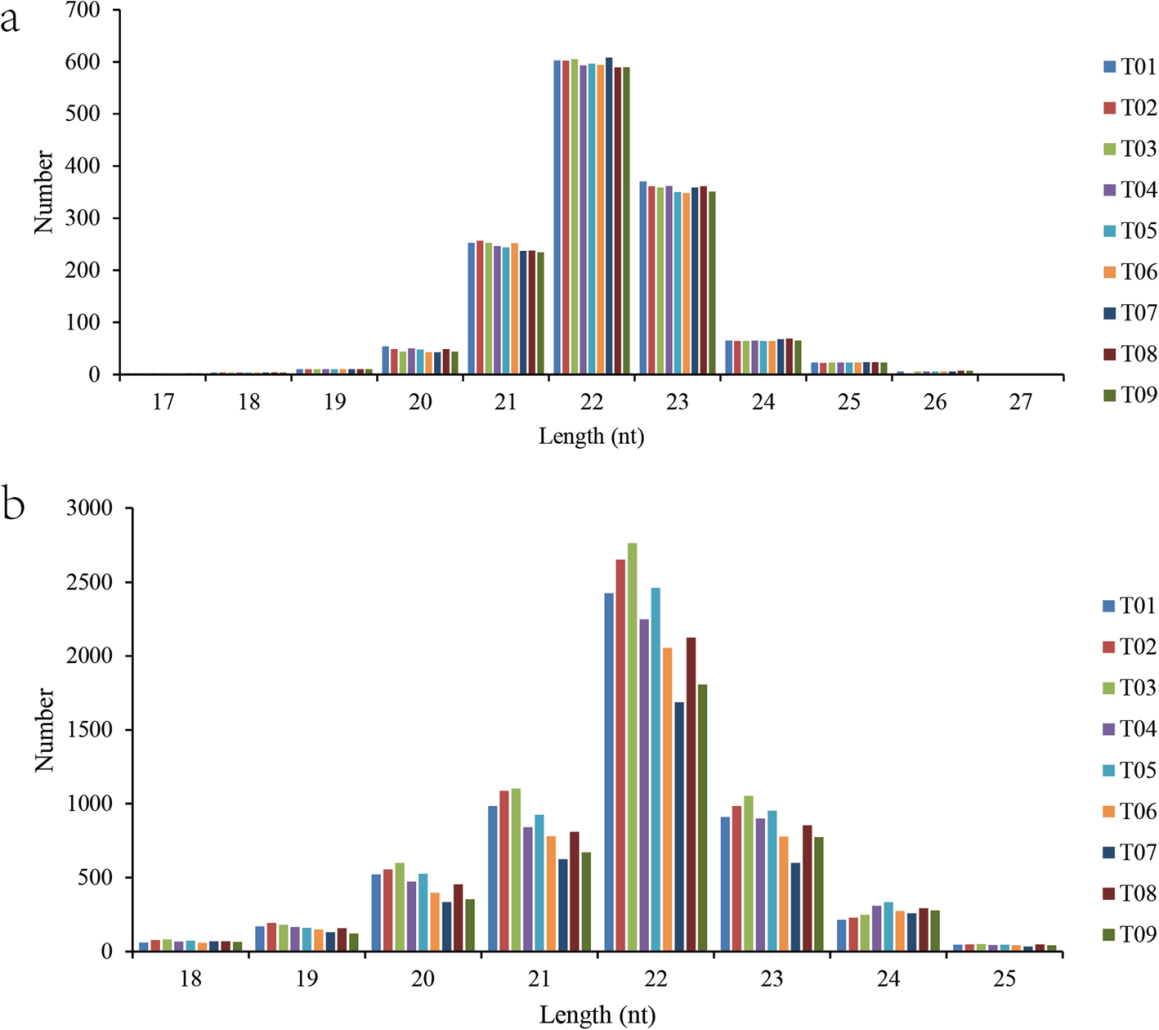
Length distribution of miRNAs in P. sinensis, **a**. known miRNAs, **b**. novel miRNAs

After discarding junk sequences, 11.7, 12.4, 14.1, 14.0, 15.1, 11.3, 11.9, 13.0 and 11.4 million clean reads were generated in the T01, T02, T03, T04, T05, T06, T07, T08 and T09 samples, respectively. The Q30% percentage per individual ranged from 95.57% to 96.64%. Reads from all libraries were annotated using the GenBank and Rfam databases. Through annotation, rRNAs, tRNAs, snRNAs and snoRNAs were separated from each other. tRNAs and rRNAs were the most abundant small RNA types (Table 3). The length distributions of miRNAs were similar among libraries in that 22 nt RNAs were the most abundant, which are typical dicer-processed miRNA products (Fig. 7). To identify the conserved miRNAs in *P. sinensis*, small RNA sequences were compared with the currently available mature miRNAs in miRBase. Finally, a total of 1 486 miRNAs in *P. sinensis* were identified as orthologues of known miRNAs, and 7 575 were predicted to be novel miRNAs in the nine microRNA libraries.

**Table 3:** Statistical analysis of sRNA sequencing data

Group	Samples	Raw reads	Length	Clean reads	% ≥ Q30	Total reads	Mapped reads
Initial Period	T01	11 739 267	19 355	11 719 912	95.57%	11 033 249	7 358 371(66.69%)
	T02	12 522 636	31 830	12 490 806	96.53%	11 739 658	8 652 966(73.71%)
	T03	14 205 890	16 744	14 189 146	96.28%	13 414 851	9 777 065(72.88%)
Middle Period	T04	14 071 713	11 539	14 060 174	96.43%	12 836 130	9 148 886(71.27%)
	T05	15 150 784	15 763	15 135 021	96.64%	14 203 102	10 259 529(72.23%)
	S06	11 408 359	12 569	11 395 790	96.46%	10 695 652	7 868 814(73.57%)
Final Period	T07	12 033 355	56 959	11 976 396	96.22%	10 207 816	7 240 751(70.93%)
	T08	13 100 186	17 184	13 083 002	96.15%	12 306 884	9 047 235(73.51%)
	T09	11 435 410	21 093	11 414 317	95.86%	10 147 317	6 858 721(67.59%)

### Analysis of differential expression of miRNAs

The DESeq program was used to determine miRNA profiles in the skin of *P. sinensis* in the incubation stage. In IP *vs.* MP, we found 277 differentially expressed miRNAs, 209 of which were upregulated and 68 of which were downregulated. In MP *vs.* FP, 158 miRNAs were differentially expressed, 139 of which were upregulated and 19 of which downregulated. Among these miRNAs, only three miRNAs (novel_miR_1414, novel_miR_1019 and novel_miR_7092) were unique in MP, and seven miRNAs, including six novel miRNAs and one miR-129 family miRNA were unique to FP. In IP *vs.* FP, there were 687 miRNAs, including 491 upregulated and 196 downregulated miRNAs in FP (Tab. S6).

### Target prediction for differentially expressed miRNAs and functional analysis

To provide clues about the possible roles of differentially expressed miRNAs, target prediction analysis was conducted by miRanda and TargetScan. The numbers of differentially expressed miRNA target genes annotated in IP *vs.* MP, MP *vs.* FP and IP *vs.* FP were 4 130, 6 576 and 1 375, respectively. GO term analysis indicated that a total of 40, 16 and 32 out of 2 756, 1 719 and 3 278 clustered GO terms were significantly enriched (*q-value* < 0.05) in IP *vs.* MP, MP *vs.* FP and IP *vs.* FP, respectively (Tab S7). Using KEGG pathway analysis, the predicted target genes in the three stages were grouped into 150, 100 and 162 pathways. Among these pathways, only ECM-receptor interaction was significantly enriched (*q-value* < 0.05) in the three groups.

### Validation of RNA-seq and miRNA-seq data by qRT–PCR

**Fig. 8:**
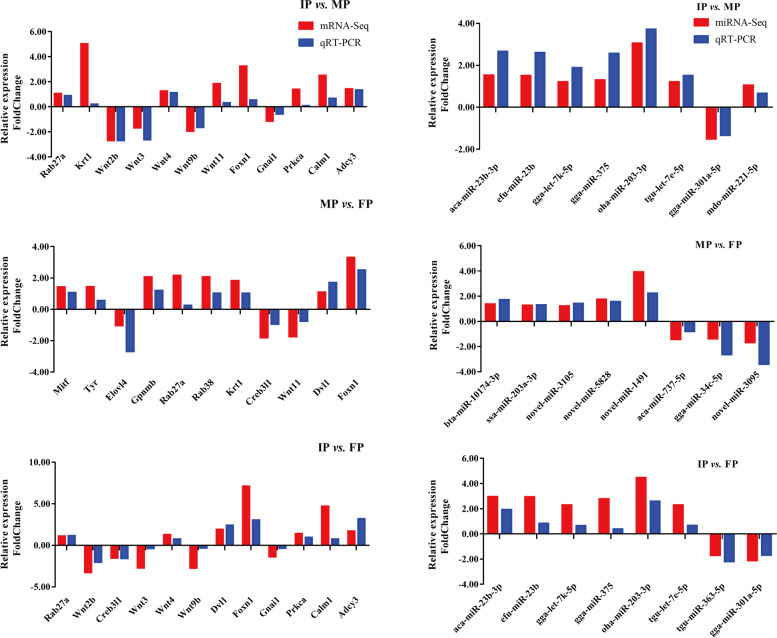
Expression levels of randomly selected mRNAs and miRNAs-seq determined by qRT–PCR. Expression levels of genes determined by qRT–PCR and RNA-seq

To evaluate the reliability of RNA-seq and miRNA**–**seq data and further validate the patterns of DEGs and DEMs, approximately 12 DEGs were randomly selected for qRT**–**PCR analysis to validate the expression patterns of the genes. To validate the expression patterns of the miRNAs, qRT**–**PCR was utilized to detect the expression of randomly selected miRNAs that were both up- and downregulated. In general, the results of qRT**–**PCR validated the results of RNA-seq and supported the reliability of identified differentially expressed miRNAs and mRNAs (Fig. 8).

### MiRNAs and mRNAs involved in pigmentation of P. sinensis

**Fig. 9:**
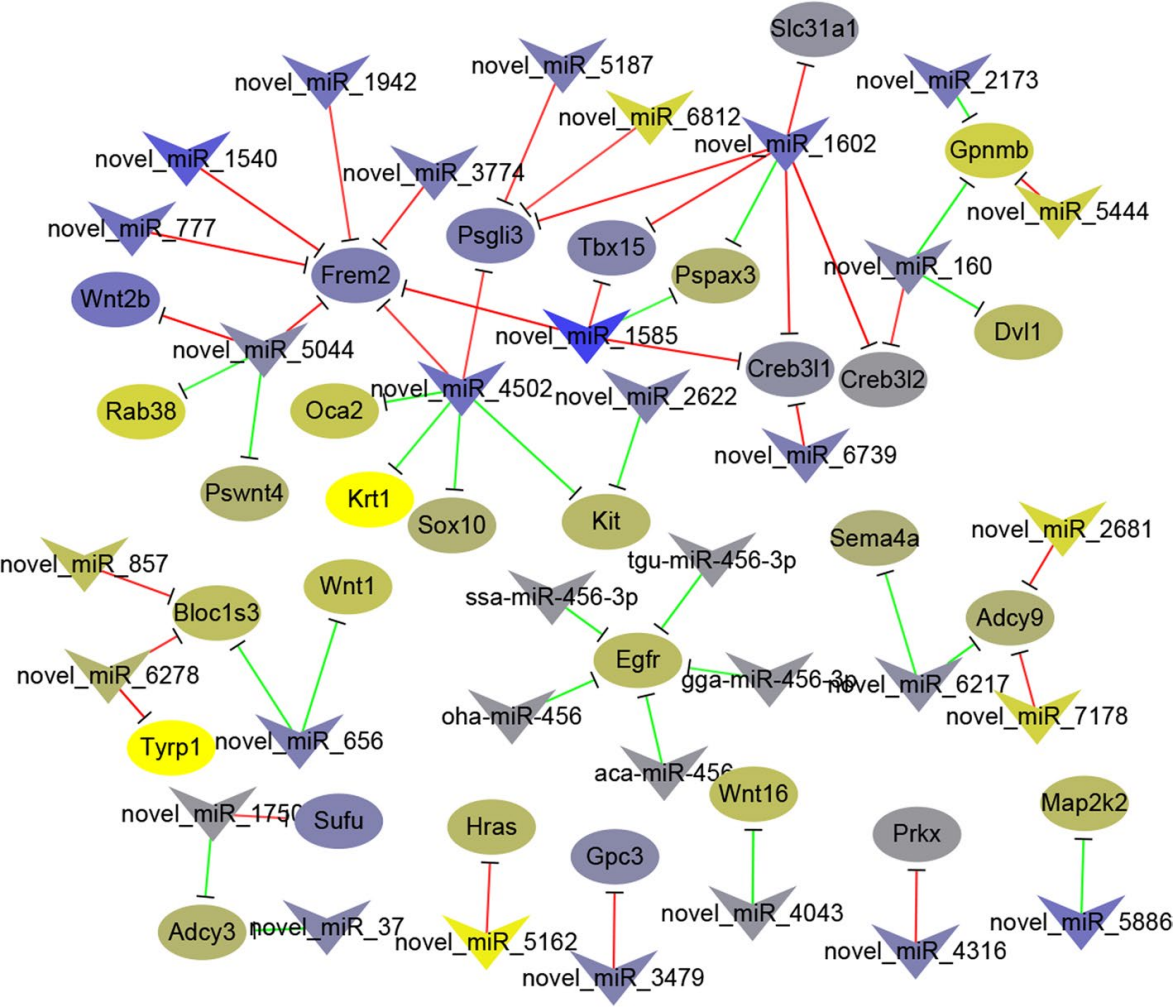
A proposed network of putative interactions between miRNAs and mRNAs in P. sinensis

**Fig. 10:**
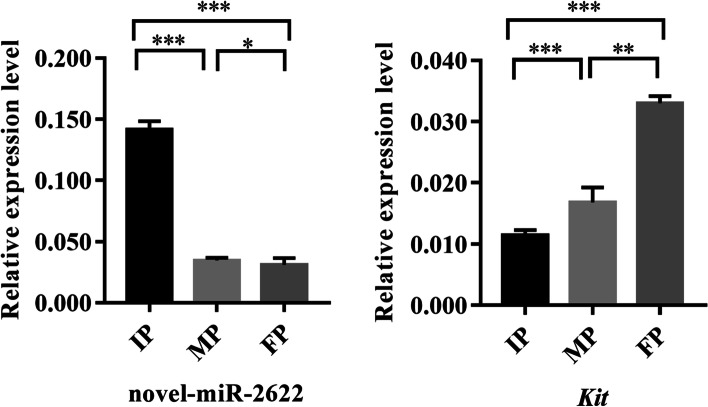
The expression of miRNA and the target genes

In the melanogenesis pathway, we found 16 differentially expressed miRNAs and 24 target genes in IP *vs.* MP. There were 6 miRNAs and 8 target genes in MP *vs.* FP, while the were 25 miRNAs and 34 target genes in IP *vs.* FP (Tab. S8). Additionally, the results of transcriptome sequencing were combined to determine the differential expression of target genes. In IP *vs.* MP, only four miRNAs and three target genes were differentially expressed at the same time. There were no pairs that showed differential expression at the same time in MP *vs.* FP. In IP *vs.* FP, 17 miRNAs and 14 target genes showed differential expression, including 11 negatively regulated pairs and 6 positively regulated pairs. Furthermore, miRNAs that regulate overlapping body colour difference genes were screened out. The results showed the greatest number of miRNA**–**mRNA regulatory pairs in IP *vs.* FP, while in MP *vs.* FP, there were no miRNA**–**mRNA regulatory pairs (Tab. S9). Based on these results, the relevant miRNA**–**mRNA network was finally generated (Fig. 9). Further screening of miRNAs that regulate body colour, confirmed that novel_miR_656 positively regulated *Wnt1*, novel_miR_1602 negatively regulated *Creb3l1*, novel_miR_5886 positively regulated *Map2k2*, novel_miR_4502 and novel_miR_2622 positively regulated *Kit*, while novel_miR_4502 negatively regulated *Sox10*, novel_miR_6278 positively regulated *Tyrp1*, novel_miR_5162 positively regulated *Hras*, novel_miR_5444 and novel_miR_160 negatively regulated *Gpnmb*, and novel_miR_7178 positively regulated *Adcy9*. Among these miRNAs, novel_miR_2622, novel_miR_6278, novel_miR_656 and novel miR_6812 were identified as orthologues of known miRNAs belonging to the miR_19, miR_138, miR_7386 and miR_129 families, respectively. Furthermore, we found that *Kit* was negatively regulated by novel_miR_2622 in qRT**–**PCR analysis (Fig. 10).

## Discussion

Skin development is a complex multifactorial process. During embryonic development, the differentiation of multipotent progenitor cells in monolayer epidermis forms epidermis and its appendages. Tokita Masayoshi et al. identified 23 stages in the embryonic development of soft-shelled turtle and described the changes in pigmentation at different developmental stages [42]. Therefore, it is feasible to conduct transcriptomic analysis on turtle embryos at different incubation stages to study pigment development. In the present study, to understand the pigmentation formation mechanism in skin, we first analysed the expression of miRNAs and mRNAs in the embryo skin transcriptome of *P. sinensis* on based RNA-seq. MiRNAs are important posttranscriptional regulators that are widely involved in a variety of biological processes in eukaryotes. Currently, deep sequencing is widely used to characterize miRNA profiles and discover novel miRNAs in a variety of organisms. A total of 8 860 novel genes were identified in the skin of turtle embryos during three incubation stages, and the identification of these novel genes makes an important contribution to the *P. sinensis* genome. Due to the lack of *P. sinensis* miRNA information in the miRNA database, the majority of these miRNAs were novel, including more than 7 000 novel miRNAs identified in this study. Therefore, this large amount of miRNA information will enrich the database and facilitate follow-up research on soft-shelled turtle miRNAs. Moreover, length analysis indicated that most sequences were 20–24 nt long, which is consistent with the typical characteristics of Dicer-processed products [43].

Melanin is the basis of different pigmentation in skin, hair, and eyes. The formation of embryo body colour involves the continuous accumulation of melanocytes. In this study, we found that the expression levels of some genes were increased significantly in all three periods. *Krt1* is a member of the keratin gene family. In alpaca skin, the expression of *Krt2* is higher in brown skin than in white skin, and the results suggested that *Krt2* functions in alpaca hair colour formation [44]. Although there have been no reports of *Krt-*related genes in the skin of soft-shelled turtle, the expression of *Krt1* in MP showed a 5.018-fold change difference from that IP and a 7.47-fold difference between FP and IP. The results indicated that *Krt1* may play an important role in the development of the embryonic skin of turtles. *Rab27a* is known to serve as a regulator of melanosome trafficking [45] and has been identified as a tumour dependency gene in melanoma [46]. Moreover, it has been reported that *Mitf* regulates the expression level of *Rab27a* [47]. *Foxn1* (*Whn, Hfn11*), a gene mutated in nude mice that encodes a transcription factor, was found to play an important role in directing keratinocytes to receive pigmented melanosomes from melanocytes [48]. *Foxn1* is not necessary in the initial stages of pigment system development. How do recipients acquire pigment in *Foxn1*-negative stages? One possibility is that they employ *Kitl* (Kit ligand, also known as *Kitlg*). *Kitl* many identify pigment recipients or activate a recipient phenotype, either in concert with *Foxn1* or as an alternative to it. In this study, we found that the expression of *Foxn1* was significantly increased in the three evaluated periods, but was not high in the initial and middle periods, while the expression of *Kitlg* was higher. These results indicated that during embryonic pigment formation in turtles, *Kitlg* may also show a synergistic effect with *Foxn1* or act as a substitute for it in the early stage of pigment formation. *Rab38* is involved in the transport of key melanogenic enzymes in vertebrates. A study reported that mutations in *Rab38* are responsible for the diluted coat colour and oculocutaneous albinism phenotype [49]. Moreover, *Mitf* is reported to be involved in the regulation of *Rab32/38* activity during *Ciona* pigment cell development [50]. *Mitf* is a key transcription factor involved in the differentiation, growth, and survival of melanocytes and can regulate more than 25 pigment genes, including *Tyr*, *Tyrp1*, and *Gpnmb* [51]. Studies indicate that the expression of *Mitf* varies in different periods [52, 53]. In addition, hormones have been reported to stimulate the expression of *Mitf*. In this study, the expression of *Mitf* increased significantly after MP. The MP sampling point was close to the sex determination period of the turtles. Perhaps the increase in sex hormones is one of the reasons for the increase in *Mitf* expression in MP. *Tyr* produces a key enzyme involved in melanin biosynthesis in melanocytes that is associated with the formation of eumelanin [54]. In addition, it is worth noting that the *Gpnmb* expression showed significant increases in the FP. *Gpnmb* has proven to be present in all stages (I-IV) of melanosomes, and is especially enriched in mature stages [55]. During FP in soft-shelled turtle embryonic development, the body surfaces are obviously darkened. The formation of body colour is the result of the accumulation and expression of various body colour genes. Among melanin-related genes, *Kit* plays a pivotal role in the melanogenesis signalling pathway, and the mutation or deletion of *Kit* can cause different hair and skin colours in mammals. It has been reported that melanogenesis can be enhanced by stem cell factor/c-kit signalling in normal human epidermal melanocytes exposed to norepinephrine [56]. It is known that *Kit* regulates cell migration, survival, proliferation, and differentiation in melanocytes [57] and interacts with *Mitf* [58]. In this study, the expression of *Kit* was significantly increased in MP but increased slowly in FP. This result indicated that the proliferation and migration of *Kit* in melanocyte was more obvious and more critical in MP. Another important gene that we identified was scavenger receptor class B, member 1 (*Scarb1*), which encodes for a lipoprotein receptor critical for the cellular uptake of carotenoids across a range of vertebrates and invertebrates [59]. A recent study demonstrated that *Scarb1* is required for carotenoid deposition in the adult xanthophores of zebrafish [60] and that *Scarb1* expression levels covary with skin carotenoid contents in lizards [61]. Similarly, higher expression of *Scarb1* is found in yellow skin of East African cichlid fish (*Tropheus duboisi*) [15]. In addition, during the embryonic development of Chinese soft-shelled turtle, xanthophores could be observed in the final period of development but not in the initial and middle periods. Additionally, the Seq-RNA results showed that the expression of *Scarb1* in FP was significantly higher than that in FP and MP. Therefore, we speculate that *Scarb1* may be related to the development of xanthophores in soft-shelled turtle.

In this study, the melanogenesis pathway was used as an example to screen the miRNAs that regulate skin pigment development of in this turtle species. Most of the miRNAs involved in regulating the melanogenesis pathway in this turtle were novel miRNAs, and the only two previously identified miRNAs were mdo-miR-221-5p and ppa-miR-152. There is convincing evidence that miR-221 is associated with several types of human cancers. Studies have reported that miR-221 is a novel biomarker of colorectal cancer, and a high level of miR-221/222 leads to increased cell invasion and poor prognosis in glioma [62, 63]. Moreover, studies have reported that *Kit* is a target of miR-221 in melanoma [64, 65]. Similarly, it has been reported that the expression of miR-152 increases during human dermal fibroblast senescence and that is overexpression is sufficient to induce cellular senescence in early-passage cells [66]. In this study, *Wnt9a* and *Adcy5* were predicted to be the target genes of miR-221 and miR-152, respectively. However, *Wnt9a* and *Adcy5* did not show differential expression. It may be that *Wnt9a* and *Adcy5* do not play a significant role in the development of pigmentation during the embryonic stage in soft-shelled turtle. Therefore, the questions of whether miR-221/152 targets and regulates *Wnt9a/Adcy5* and how *Wnt9a/Adcy5* is regulated will require further verification. In this study, through the analysis of novel miRNA families, several novel miRNA families that regulate body colour genes were found. *Blocls3* was regulated by novel_miR_656 and novel_miR_6278, and these two novel miRNAs belonged to the miR-7386 family and miR-138 family, respectively. Novel_miR_6278 also positively regulated *Tyrp1*. Novel_miR_2622, belonging to the miR-19 family, was found to negatively regulate the *Kit* gene. Similarly, novel_miR_2173 also belongs to the miR-19 family and negatively regulates *Gpnmb*. In non-small cell lung cancer cells, *Gpr124* was proven to be a direct target of miR-138-5p, and its expression was suppressed by miR-138-5p [67]. In a study by Wang et al., miR-138-5p was predicted to play important roles in regulating the pigmentation process in *Rad tilapia* [68]. In another study, we showed that *Tyr* is also one of the target genes of miR-138-5p. Furthermore, we found that miRNAs exerted both positive and negative regulatory effects on mRNAs. In general, miRNAs can downregulate expression of targeted genes [69]. Some studies have reported that miRNAs may also mediate the enhancement of translation, which may be caused by the different action sites of miRNAs and mRNAs. MiRNA-10a enhances ribosomal protein mRNAs and translation by binding to their 5’UTR [70]. mRNA 3’ UTRs expressed in proliferating cells are conservatively shortened, resulting in a decrease in the target sites of miRNAs, thereby avoiding the negative regulatory effect of miRNAs [71]. Therefore, the positive regulatory relationships screened out in this study also deserve further study.

MiR-19, which belongs to the miR-17 ~ 92 cluster, is usually reported to be involved in the proliferation and differentiation of tumour cells [72], but there have been few relevant reports in the context of skin pigmentation. In addition, miR-19 has been reported to potentiate NF-κB activity in inflammation [73]. There are also reports that toll-like receptor 9 and interleukin 10 protect melanogenesis through NF-κB activation [74, 75]. In the melanogenesis pathway, *Kit* acts as an upstream gene of *Mitf*; it regulates melanogenesis via the activation of *Mitf,* which controls differentiation and is responsible for upregulating the transcription of pigmentation enzymes (Tyr, Tyrp1 and Dct). *Kit* and *Mitf* exhibit complex interactions: *Mitf* is required to maintain kit expression in melanoblasts (melanocyte precursors), and *kit* signalling modulates *Mitf* activity and stability in melanocytes [76]. In this study, the expression levels of *Kit* and *Mitf* showed a complementary relationship, in which they together maintained the proliferation and differentiation of melanocytes, and *Mitf* acted as a core gene regulating the expression of downstream genes and promoting melanin synthesis. However, there are no reports clearly showing that miR-19 is involved in body colour regulation. In this study, the sequencing results and qRT‒PCR results verified that novel_miR_2622 shows a negative regulatory relationship with *Kit*. *Kit* has been reported to be involved in melanin proliferation, and miR-19 has been reported to be involved in regulating cell proliferation. Therefore, we speculate that novel_miR_2622 negatively regulates the expression of *Kit*, thereby promoting the expression of downstream body colour genes, affecting melanin synthesis, and promoting the formation of body colour.

## Conclusions

To provide insights into the mechanism of pigmentation in *P. sinensis.* We combined mRNA-seq with miRNA-seq, and identified 2 867 DEGs and 277 DEMs in IP *vs.* MP, 1 840 DEGs and 158 DEMs in MP *vs.* FP, 4 290 DEGs and 687 DEMs in IP *vs.* FP. The differentially expressed genes were significantly enriched in linoleic acid metabolism, ECM-receptor interaction and drug metabolism—cytochrome P450. And in the melanogenesis, cell adhesion molecules (CAMs) and the ErbB signalling pathway. Subsequently, 72 genes, 25 miRNAs and 17 corresponding target genes that may be involved in body colour regulation were obtained. Compared with previous studies, these DE mRNAs and miRNAs are likely involved in pigmentation. The quantification of novel_miR_2622 and *Kit* revealed a negative regulatory relationship, indicating that novel_miR_2622 may participate in embryonic pigmentation in *P. sinensis* by negatively regulating the expression of *Kit*. Taken together, this work represents the first attempt to use RNA-seq and miRNA-seq technology to study the pigmentation of Chinese soft-shelled turtle in embryonic development stages, and the results provide novel insights into the genetic mechanism of pigmentation and will contribute to future research.

## Materials and methods

### Sample collection

All fertilized eggs of Chinese soft-shelled turtles used in this study were obtained from the HeZhou breeding centre (Changde, Hunan, China). The temperature was maintained at 30 °C during incubation. When hatched at 20 days, the whole embryo was milky white, and few melanocytes could be observed on the head or body surface. With the prolongation of the incubation time, the number of melanocytes on the surface of the embryos was increased on the 30^th^ day of incubation, and the whole-body colour was black on the 40^th^ day, similar to that of the hatchlings. Therefore, the 20^th^, 30^th^ and 40^th^ days of incubation were defined as the initial (T1/T2/T3), middle (T4/T5/T6) and final (T7/T8/T9) pigment formation periods, respectively. Three normally developed fertilized eggs were selected for sampling from each stage. After opening the eggshell, the embryo was removed with forceps and placed in a culture dish, and the skirt was removed with a dissecting needle and forceps. Skirts tissue samples were immediately collected in RNA protection solution (Takara, 9750, Beijing). After overnight treatment at 4 °C, the samples were stored at -80 °C until use.

### RNA isolation and library preparation for RNA sequencing

Total RNA was isolated using TRIzol Reagent (Invitrogen, USA) following the manufacturer’s instructions. RNA purity and concentrations were measured using a NanoDrop ND-2000 (Thermo Fisher Scientific, Wilmington, MA, USA). The Agilent Bioanalyzer 2100 system (Thermo Fisher Scientific, MA, USA) was utilized to assess the integrity of the obtained RNA. Total RNA with good quality was used for further experiments. A total amount of 1 µg RNA per sample was used for RNA library preparation. Sequencing libraries were generated using the NEBNext UltraTM RNA Library Prep Kit for Illumina (NEB, USA) following the manufacturer’s protocol and index codes were added to identify sequences in each sample. The nine obtained cDNA libraries were sequenced on the Illumina HiSeq 4000 platform and paired-end reads were generated. Library construction and RNA-Seq were performed at Beijing BioMarker Technologies (Beijing, China) in accordance with the institute’s protocols.

### Analysis of RNA-Seq data

Raw sequence data were transformed by base calling into sequence data and stored in fastq format. Clean data were obtained by removing low-quality reads. All downstream analyses were based on clean high-quality data. These high-quality clean reads were then mapped to the genome sequence of *Pelodiscus sinensis* genome using TopHat2. Cufflinks was then used to splice the mapped reads, and then the reads were compared with the annotation of the reference genome to identify new transcripts. Gene expression levels were estimated based on the fragments per kilobase of transcript per million fragments (FPKM) mapped method in different samples.

### Differentially expressed genes (DEGs) and functional enrichment analysis

The fragments per kilobase of exon per million fragments mapped values of each gene were calculated based on the length of the gene and the read count mapped to the gene. DEGs among the three groups were analysed using the DESeq R package. DESeq provides statistical routines for determining differential expression in digital gene expression data using a model based on the negative binomial distribution. The resulting *P* values were adjusted using Benjamini and Hochberg’s approach for controlling the false discovery rate (FDR < 0.01). Genes expressions with an absolute value of the expression fold change (FC) ≥ 2 and an adjusted *p-* value < 0.01 according to by DESeq were assigned as differentially expressed, and were divided into lists of upregulated and downregulated transcripts. The RNA-seq data are publicly available in the NCBI database under accession number PRJNA701407.

Gene Ontology (GO) enrichment analysis of the DEGs was implemented with the GOseq R package-based Wallenis noncentral hypergeometric distribution for adjusting gene length bias in DEGs. GO terms with a corrected *p-* value < 0.05 were considered significantly enriched by DEGs. GO analysis was performed to retrieve biological process, molecular function, and cellular component information to obtain Blast2GO annotations for all genes related to the formation of pigmentation. The Kyoto Encyclopedia of Genes and Genomes (KEGG) was used to assign and predict the functions and metabolic pathways of the DEGs [77]. KOBAS software was used to test the statistical enrichment of DEGs in KEGG pathways.

### RNA isolation, small RNA library preparation and sequencing

The total RNA extraction method was the same as that employed for RNA-seq. A total of 1.5 µg of total RNA was used for the construction of libraries using the NEB Next Multiplex Small RNA Library Prep Kit (Illumina Inc., San, Diego, CA, USA) according to the manufacturer’s protocol. The purified RNAs were ligated with 3’ and 5’ adapters for Illumina processing. Reverse transcription followed by PCR was used to create cDNA constructs based on the small RNAs ligated with 3’ and 5’ adapters. DNA fragments of 140–160 bp in length were purified. The cDNA library was recovered for Illumina sequencing library preparation. The small RNA library inserts were 18–30 bp, and the library was sequenced on an Illumina HiSeq 2000 platform.

### miRNA identification

The original sequences obtained by sequencing contains linker sequences or low-quality sequences. To ensure the accuracy of information analysis, quality control of the original data is required to obtain high-quality sequences (clean reads). The quality control standards for the original sequence were as follows: (1) for each sample, read with > 20% base < Q 30 were removed; (2) reads with an unknown base N content greater than or equal to 10% were remove; (3) reads without 3’ adapters were removed; (4) 3’ adapters sequences were cut off; and (5) reads < 18 bp or > 30 bp were removed. The remaining clean reads were searched against the Silva database, GtRNAdb database, Rfam database and Repbase database to filter ribosomal RNA (rRNA), transfer RNA (tRNA), small nuclear RNA (snRNA), small nucleolar RNA (snoRNA) and other ncRNAs and repetitive sequences to obtain unannotated reads containing miRNA. Bowtie software was used to align the unannotated reads with the *P. sinensis* reference genome to obtain position information from the reference genome. The unannotated reads containing small regulatory RNAs were processed for miRNA identification by using the miRBase (v22) database. The miRDeep2 software package was used based on the information on the read distribution in precursor sequences, and precursor structure energy information (RNA fold) was scored using a Bayesian model to prediction new miRNA [78].

### Expression analysis, target gene prediction, and GO/KEGG enrichment analysis

The expression levels of total miRNAs were estimated based on transcripts per million (TPM) values. Differential expression analysis of samples was performed using the DEGseq package in R. The miRNAs with a (FC) ≥ 2 and *q*-value < 0.01 were identified as significantly differentially expressed. The putative target genes were predicted with two computational target prediction algorithms, miRanda and TargetScan, and the overlapping genes between the two algorithms were regarded as the final target genes. To reveal the possible functions of the target genes, GO terms and KEGG pathways were used for enrichment analysis. Cytoscape was used to visualize the interaction between miRNAs and mRNAs.

### Confirmation of mRNAs and miRNAs using real-time quantitative polymerase chain reaction (qRT‒PCR) validation

To validate the repeatability and reproducibility of DEGs obtained from RNA-seq and DEMs from miRNA-seq data, mRNAs and miRNAs were randomly selected and quantified by qRT**‒**PCR. Primers were designed using Primer Premier 6.0 (Table S10). The qRT**‒**PCR was performed using the StepOnePlus™ system (Thermo). Total RNA was reverse transcribed into cDNA by using the PrimeScript™ RT reagent Kit with gDNA Eraser (Takara, Japan). The mRNA qRT**‒**PCR procedure was conducted according to the following conditions: 95 °C for 30 s for denaturation, followed by 40 cycles of 95 °C for 5 s and 60 °C for 30 s. The qRT**‒**PCR was conducted using 5 µL TB Green™ Premix Ex Taq ^TM^II(2X) (Takara, Japan), 0.4 µL forward and reverse primers, 0.2 µL ROX reference dye (50X), 1 µL c DNA and 3 µL ddH2O. The total reaction volume was 10 µL. The *Gapdh* gene and *β-actin* were used as internal controls. Total small RNAs were extracted from skins using the miRcute miRNA Isolation Kit (Tiangen, Beijing, China). Poly (A) tail addition and the reverse transcription of 2 µg RNA were performed using the miRcute miRNA First- Strand cDNA synthesis Kit (Tiangen, Beijing, China). qRT**‒**PCR was then performed using the miRcute Plus miRNA qPCR Kit (SYBR Green) (Tiangen, Beijing, China). qPCR was conducted in 10 µL of PCR solution containing 1 µL cDNA, 5 µL miRcute miRNA premix (2X), 1 µL of 50 × ROX Reference Dye, 0.2 µL forward primer, 0.2 µL reverse primer, and 2.6 µL ddH2O according to the following program: 95 °C for 15 s for denaturation, followed by 40 cycles of 95 °C for 20 s, 60 °C for 15 s and 72 °C for 15 s. The U6 was used as the internal control for the normalization of expression levels. All reactions were performed with three biological replicates. The relative expression levels of the target genes were calculated with the 2^−△△ct^ method.

## Supplementary Information

**Additional file 1:** **Tab. S1.** Pairwise comparison of different experission genes in intial period, middle period and final period. **Tab. S2.** Differentially expressed genes in veen diagram. **Tab. S3.** GO enrichment analyses of DEGs from comparisons of IP vs. MP, MP vs. FP and IP vs. FP. **Tab. S4.** The differentially expressed genes in the melanogenesis pathway among three period of embryo incubation of P.sinensis. **Tab. S5.** List of candidate genes for pigmentation  differentially significantly expressed in three incubation stages of Chinese soft-shelled turtle (P. sinensis). **Tab S6.** List of differentially expressed miRNAs of P.sinensis in different incubation stages. **Tab. S7.** The enriched of GO term analysisof target genes. **Tab. S8.** The Differential expression of miRNA and target genes in Melanogenesis pathway pathway in embryon of P.sinensis. **Tab. S9.** Differentially expressed miRNAs and genes related to body color. **Table S10.** qRT-PCR primer information for mRNA and miRNA.

## Data Availability

The datasets supporting the conclusions of this article are available as follows: Sequence reads from the RNA-Seq experiment are available from the NCBI sequence read archive under the accession number PRJNA701407(https://www.ncbi.nlm.nih.gov/bioproject/701407).
